# Neurocognitive outcomes in pediatric brain tumors after treatment with proton versus photon radiation: a systematic review and meta-analysis

**DOI:** 10.1007/s12519-023-00726-6

**Published:** 2023-05-08

**Authors:** Álvaro Lassaletta, Javier S. Morales, Pedro L. Valenzuela, Borja Esteso, Lisa S. Kahalley, Donald J. Mabbott, Soumya Unnikrishnan, Elena Panizo, Felipe Calvo

**Affiliations:** 1 Radiation Oncology Department, Clínica Universidad de Navarra, Calle Marquesado de Santa Marta 1, 28027 Madrid, Spain; 2 Pediatric Neuro-Oncology Unit, Hospital Infantil Universitario Niño Jesús, Madrid, Spain; 3 MOVE-IT Research Group, Department of Physical Education, Faculty of Education Sciences, University of Cadiz, Cádiz, Spain; 4 Biomedical Research and Innovation Institute of Cádiz (INiBICA) Research Unit, Puerta del Mar University Hospital, University of Cádiz, Cádiz, Spain; 5 Physical Activity and Health Research Group (PaHerg), Research Institute of the Hospital 12 de Octubre (‘imas12’), Madrid, Spain; 6 Systems Biology Department, University of Alcalá, Madrid, Spain; 7 Clinical Neuropsychology Unit, Psychiatry and Clinical Psychology Department, Hospital Infantil Universitario Niño Jesús, Madrid, Spain; 8 Baylor College of Medicine, Houston, TX USA; 9 Texas Children’s Hospital, Houston, TX USA; 10 The Hospital for Sick Children, Toronto, ON Canada; 11 The University of Toronto, Toronto, ON Canada; 12 University of California School of Medicine, San Diego, La Jolla, CA USA

**Keywords:** Brain health, Childhood cancer, Intelligence quotient, Radiotherapy

## Abstract

**Background:**

Advances in cancer treatments, particularly the development of radiation therapy, have led to improvements in survival outcomes in children with brain tumors. However, radiation therapy is associated with significant long-term neurocognitive morbidity. The present systematic review and meta-analysis aimed to compare the neurocognitive outcomes of children and adolescents with brain tumors treated with photon radiation (XRT) or proton therapy (PBRT).

**Methods:**

A systematic search was conducted (PubMed, Embase, Cochrane, and Web of Science from inception until 02/01/2022) for studies comparing the neurocognitive outcomes of children and adolescents with brain tumors treated with XRT *vs*. PBRT. The pooled mean differences (expressed as Z scores) were calculated using a random effects method for those endpoints analyzed by a minimum of three studies.

**Results:**

Totally 10 studies (*n* = 630 patients, average age range: 1–20 years) met the inclusion criteria. Patients who had received PBRT achieved significantly higher scores (difference in Z scores ranging from 0.29–0.75, all *P* < 0.05 and significant in sensitivity analyses) after treatment than those who had received XRT for most analyzed neurocognitive outcomes (*i.e.*, intelligence quotient, verbal comprehension and perceptual reasoning indices, visual motor integration, and verbal memory). No robust significant differences (*P* > 0.05 in main analyses or sensitivity analyses) were found for nonverbal memory, verbal working memory and working memory index, processing speed index, or focused attention.

**Conclusions:**

Pediatric brain tumor patients who receive PBRT achieve significantly higher scores on most neurocognitive outcomes than those who receive XRT. Larger studies with long-term follow-ups are needed to confirm these results.

**Supplementary Information:**

The online version contains supplementary material available at 10.1007/s12519-023-00726-6.

## Introduction

Brain tumors are the second leading cause of cancer in children and adolescents and the leading cause of cancer death in this population [1]. The first attempts to use X-ray therapy (roentgen therapy) were made by Percival Bailey and Harvey Cushing in the 1930s. They found that a subset of patients at autopsy had no evidence of medulloblastoma but rather radiation necrosis [2]. This led other groups to explore radiation therapy as a treatment after surgery in pediatric brain tumors [2]. Later, Paterson in the 1950s began administering craniospinal radiation to pediatric patients with medulloblastoma [3]. These achievements raised the survival outcomes of children with brain tumors but also increased the neurocognitive sequalae of these patients. Since then, evidence has shown that pediatric brain tumor survivors treated with cranial radiotherapy have a remarkable risk of neurocognitive impairment, not only in global intellectual functioning [*e.g*., full-scale intelligent quotient (FSIQ)] but also in specific cognitive domains such as executive function, attention, memory, processing speed, and fine motor control [4–11]. In fact, there is meta-analytical evidence confirming the neurocognitive decline associated with photon radiation (XRT) in children and adolescents with brain tumors [7].

Many strategies have been developed to decrease the neurocognitive side effects of these children, including the use of chemotherapy to reduce radiation doses in many pediatric brain tumors [12] and the reduction in the radiation boost volume [13]. However, the greatest advances have probably been made in radiotherapy techniques, which have sought to deliver intended doses to the target tumor while reducing the exposure of surrounding healthy brain tissue, with the goal of decreasing radiation-induced long-term complications.

Proton therapy (PBRT) is becoming widely used in high-income countries across the world. It was first available in the US for children at the Harvard Cyclotron after 1974. Children with central nervous system (CNS) tumors were treated in 1992 at Loma Linda University Medical Center (CA, USA) [14]. However, proton therapy started to become more available for children in 2000 (*e.g*., Boston 2000, MD Anderson 2006) [15]. The potential advantage of PBRT over XRT is the ability to reduce the exposure of healthy tissue around the target area, with the potential to reduce its deleterious effects on neurocognitive outcomes. However, few studies have compared the effects of XRT and PBRT on neurocognitive outcomes in pediatric patients with brain tumors. To date, these studies have been retrospective, as it is challenging to conceive a study for children with brain tumors randomized to photons or protons due to ethical concerns [16], as dosimetric studies have repeatedly shown the superiority of PBRT, and clinical studies have demonstrated benefits including lower late endocrine deficits, reduced radiation-induced neoplasia, and cardiac mortality [17–19]. In addition, there is a significant cost difference between XRT and PBRT, which makes these results very important. Proton therapy may be justified not only because of the already known dosimetric benefits [20] but also in neurocognitive outcomes and later quality of life.

In this context, the present systematic review and meta-analysis aimed to compare the neurocognitive outcomes of children and adolescents with brain tumors treated with XRT or PBRT.

## Methods

This review is registered in PROSPERO (CRD42020204102). We followed the guidelines of the Preferred Reporting Items for Systematic Reviews and Meta-analyses [21].

### Data sources and search strategies

Two authors (AL, JSM) independently conducted a systematic search in the electronic databases PubMed, Web of Science, Embase and Cochrane for relevant articles written in English (from inception to February 1, 2022) using the following search strategy: (proton) AND (child* OR pediatric OR pediatric OR infant OR adolescen*) with no filters for language, article type or any other filter. An example of the search is available in Supplementary Table 1. The search was supplemented by a manual review of reference lists from included studies and review articles to find additional studies on the subject.

### Study selection

Citations were first retrieved and preliminarily screened by title and abstract, and the full texts of those studies that met the inclusion criteria were assessed. Disagreements between authors were resolved through consensus or after consultation with a third reviewer (PLV). Studies were eligible for inclusion if they met the following criteria: (1) included one group of survivors of pediatric brain tumors treated with PBRT; (2) compared with a control group of survivors of pediatric brain tumors treated with XRT; and (3) assessed neurocognitive outcomes.

### Data extraction

The following data were extracted from each study: number of participants in each group, characteristics of the participants, socioeconomic status, cancer and treatment characteristics, cognitive domain measures, and neurocognitive-related results. Data were extracted, when available, as the mean and standard deviation (SD) for each study group at both baseline and postintervention, although all studies provided only postintervention data. When data were provided using other measures of dispersion [*e.g.*, 95% confidence interval (CI)], the required information was estimated following the guidelines reported elsewhere [22]. When the standard error was reported instead of the SD, the latter was obtained through the formula of Altman and Bland [23]. Endpoint data were transformed into *Z* scores [mean (M) = 0; standard deviation = 1] from each norm test value to homogenize them and enable comparisons between tests obtained using different types of measurement. We also contacted the authors of four studies [24–27] because the required data were not reported. The authors of three studies [24, 25, 27] provided the required information.

### Outcomes

Tests used for general neurocognitive abilities measures included the intelligent quotient (IQ) (Wechsler Scales of Intelligence, Bayley Scales of Infant and Toddler Development, Stanford-Binet Intelligence Scales, Reynolds Intellectual Assessment Scales, Woodcock-Johnson Tests of Cognitive Ability, Leiter International Performance Scale, Differential Abilities Scales and Raven’s Progressive Matrices), as well as Wechsler Intelligence indices for verbal comprehension index, verbal quotient (verbal IQ), perceptual reasoning index, fluid reasoning index, working memory index, and processing speed index. Wechsler indices were merged; specifically, verbal comprehension index and verbal IQ scores were analyzed as verbal comprehension scores, and perceptual reasoning and fluid reasoning indices were analyzed as perceptual reasoning scores. For specific neurocognitive abilities, tests examined visual motor integration (Beery-Buktenica Developmental Test of Visual Motor Integration), verbal memory (California Verbal Learning Test Children’s Edition, and California Verbal Learning Test Second Edition delayed recall test, Wide Range Assessment of Memory and Learning-2, and Children’s Memory Scale delayed story memory test), nonverbal memory (A Developmental Neuropsychological Assessment—Second Edition and Wechsler Memory Scales-IV memory for designs tests), verbal working memory (Wechsler Digit Span subtest) and focused attention/information processing speed (Wechsler Coding subtest). The tests carried out in each study are specified in Table 1.

**Table 1:** Main characteristics of the studies included in the systematic review and meta-analysis

Authors	Sample demographics (*n*, sex, age)	Socioeconomic status (mean ± SD)	Date range of treatment administration	Main cancer and treatment characteristics	Cognitive domain measures (test)	Main results	NOS quality score (highest possible = 10 stars)
Ali et al. [26]	PBRT: *n* = 41 (16 female), 2.1 ± 1.0 (0.5–4.7) y XRT: *n* = 37 (11 female), 1.8 ± 1.1 (0.4–4.6) y	PBRT: 35.0 ± 13.1 XRT: 35.6 ± 12.7 Socioeconomic status assessed through the Barratt Simplified Measure of Social Status (scores range from 8 to 66 with higher scores indicative of higher socioeconomic status)	November 2007 to May 2017	Age at diagnosis, mean ± SD (range): PBRT: 2.0 ± 1.1 (0.4–4.6) y XRT: 1.8 ± 1.1 (0.4–4.5) y Tumor histology: Medulloblastoma, ependymoma (anaplastic), ependymoma, atypical teratoid rhabdoid tumor, primitive neuroectodermal tumor, glioma (high grade), other Tumor location: Infratentorial Supratentorial	General cognitive skills: IQ (Bayley-III and SB-5) Executive function: Working Memory (BRIEF-P) Behavioral, and adaptive functioning: Attention and adaptive skills (BASC-2 and Bayley-III)	No differences in cognitive outcomes based on treatment modality	Total: 7 stars Selection: *** Comparability: ** Outcome: **
Child et al. [32]	PBRT: *n* = 58 (23 female), 14.0 (6.1–23.5) y XRT: *n* = 30 (7 female), 16.5 (8.5–31.4) y	N/R	PBRT: 2007 to 2013 XRT: 2001 to 2006	Age at diagnosis, mean (range): PBRT: 7.0 (1.1–16.1) y XRT: 6.5 (0.9–18.0) y Total radiation dose, median (range): Focal: PBRT (*n* = 30): 50.4 (45.0–59.4) y XRT (*n* = 13): 54.0 (48.6–59.4) y CSI: PBRT (*n* = 28): 54.0 (45.0–55.8) y XRT (*n* = 17): 54.0 (30.6–55.8) y Total radiation CSI dose, median (range): PBRT (*n* = 28): 23.4 (18.0–36) y XRT (*n* = 17): 30.0 (21.0–39.6) y Time from RT to last assessment, mean (range): PBRT: 6.0 (1.2–11.1) y XRT: 9.0 (4.0–15.3) y Tumor histology: Medulloblastoma/PNET, glioma, ependymoma, craniopharyngioma, germ cell tumor, others Tumor location: Infratentorial Supratentorial Both	General cognitive skills: FSIQ, verbal comprehension, perceptual reasoning, working memory, and processing speed indices (WAIS-IV and WISC-V) Motor functions: Fine motor (Grooved pegboard) Memory/learning: Verbal learning and memory (CVLT-C and CVLT-II), visual learning and memory (NEPSY-II and WMS-IV) Attention: d’, omissions, and comissions (CPT-II) Executive function: Switching–verbal and graphomotor–, and inhibition/switching (DKEFS) Academics: Reading fluency, math fluency, and writing fluency (WJ-III COG)	↑ Inhibition/switching with focal PBRT compared with XRT ↑ Inattention/impulsivity with craniospinal PBRT compared with XRT ↑ Survivors treated with focal PBRT performed overall better than those treated with craniospinal PBRT Results after adjusting for shunt, interval between RT and evaluation, CSI and total radiation dose, posterior fossa boost, and Lansky/Karnofsky score	Total: 7 stars Selection: *** Comparability: ** Outcome: **
Eaton et al. [33]	PBRT: *n* = 17 (6 female), 8.5 ± 4.1 y XRT: *n* = 20 (10 female), 8.5 ± 4.1 y	N/R	2000 to 2009	Age at diagnosis, mean (range): PBRT: 7.3 (3.4–20.0) y XRT: 8.1 (4.5–16.6) y Total radiation CSI dose, median (range): PBRT: 23.4 (18.0–27.0) XRT: 23.4 (18.0–26.4) Total dose to primary: PBRT: 54–55.8 Gy (*n* = 17) XRT: 54–55.8 Gy (*n* = 19) > 55.8 Gy (*n* = 1) Tumor histology: Medulloblastoma	General cognitive skills: FSIQ (Wechsler Scales-70% of sample-, DAS-25%- and RIAS-5%-), verbal comprehension, perceptual reasoning, working memory, and processing speed indices	↑ FSIQ ↑ Verbal comprehension ↑ Perceptual reasoning	Total: 6 stars Selection: ** Comparability: ** Outcome: **
Gross et al. [34]	PBRT: *n* = 58 (21 female), 8.5 (5.8–11.8)^a^ y XRT: *n* = 67 (30 female), 7.4 (4.6–11.0)^a^ y	PBRT: 7.4 (5.6–9.2)^a^ Low (< 5): 14% Average (5 and < 9): 58% High (≥ 9): 28% XRT: 6.2 (5.1–8.0)^a^ Low (< 5): 27% Average (5 and < 9): 58% High (≥ 9): 15% Socioeconomic status defined as median household income by patient zip code/10,000	1998 to 2017 (for the sensitivity analysis they excluded patients in the XRT cohort treated before 2004)	Tumor histology: Craniopharyngioma, medulloblastoma/PNET, ependymoma, germinoma, glioma, other Tumor location: Cerebral hemispheres Midline/Thalamic Posterior Fossa Time from completion of RT to last assessment, median (IQR): 3.2 (1.8–4.7) y CSI: PBRT: None (*n* = 33) 23.4 Gy or GY (RBE) (*n* = 13) 36 Gy or Gy (RBE) (*n* = 12) XRT: None (*n* = 26) 23.4 Gy or GY (RBE) (*n* = 24) 36 Gy or Gy (RBE) (*n* = 17)	General cognitive skills: FSIQ, general ability, verbal comprehension, nonverbal reasoning, performance iq, and processing speed indices, and digit span test, (Wechsler: WAIS-III/WAIS-IV, WISC-IV/WISC-V and WASI/WASI-II) Perceptual-motor functions: visual motor integration (Beery-Buktenica Developmental Test) Memory/learning: verbal learning and memory (WRAML2 and CMS) Attention: coding test (Wechsler) Executive function: digit span (Wechsler) Social cognition: ABAS social domain (ABAS-II/ABAS-3) Academics: reading (coding) and written calculation (WIAT-III and WJ-III/IV ACH) Adaptive functions: parent-reported general adaptive composite, abas conceptual domain, and ABAS practical domain (ABAS-II/ABAS-3)	Univariate analyses: ↑ FSIQ/ general Ability ↑ Verbal reasoning ↑ Performance IQ ↑ Processing speed ↑ Visual motor integration ↑ Digit span ↑ Reading/decoding ↑ Written calculations Multivariate analysis: ↑ FSIQ/ general ability ↑ Processing speed	Total: 5 stars Selection: ** Comparability: * Outcome: **
Kahalley et al. [25]	PBRT: *n* = 90 (36 female), 9.2 ± 4.1 (1.7–18.2) y XRT: *n* = 60 (27 female), 8.1 ± 3.9 (1.2–18.0) y	PBRT: 12.6 ± 11.6 XRT: 15.2 ± 11.3 Socioeconomic status reported as the percent of households in poverty within the home zip code of a patient	PBRT: 2007 to 2012 XRT: 2002 to 2007	Age at diagnosis, mean ± SD (range): PBRT: 8.6 ± 4.3 (1.1–17.8) XRT: 7.8 ± 4.0 (0.6–17.9) Total radiation dose, median (range): PBRT: 54 (30–60) Gy XRT: 54 (31–59) Gy TB boost dose, median (range): PBRT: 54 (30–56) Gy XRT: 56 (44–56) Gy Total radiation CSI dose, median (range): PBRT: 23 (21–40) Gy XRT: 23 (21–40) Gy Tumor histology: Glioma, medulloblastoma/PNET, ependymoma, germ cell tumor, other Tumor location: Infratentorial Supratentorial	General cognitive skills: IQ (Wechsler Scales of Intelligence-70.8%-, Leiter International Performance Scale-18.7%-, and WJ COG-10.5%-)	↑ IQ Results after adjusting for all demographic and medical variables that the authors identified as significantly different between groups	Total: 5 stars Selection: ** Comparability: * Outcome: **
Kahalley et al. [24]	PBRT: *n* = 37 (11 female), 9.3 ± 3.0 (3.7–14.5) y XRT: *n* = 42 (15 female), 9.5 ± 3.1 (4.8–17.8) y	N/R	2007 to 2018	Age at diagnosis, mean ± SD (range): 8.6 ± 3.0 (3.5–15.3) Total dose to TB + margin, median (range): 56 (51–59) Gy Total radiation CSI dose, median (range): 23 (15–40) Gy Tumor histology: Medulloblastoma	General cognitive skills: global IQ, verbal reasoning, perceptual reasoning, working memory, and processing speed indices (WISC-IV/V-63.2%-, WJ-III COG-36.1%-, and SB-5–0,7%-) Attention: coding test (Wechsler) Executive function: digit span test (Wechsler)	↑ IQ ↑ Perceptual reasoning ↑ Working memory Results after adjusting for total boost dose, boost margin, posterior fossa syndrome, and test measure	Total: 6 stars Selection: ** Comparability: ** Outcome: **
Peterson et al. [30]	PBRT: *n* = 22 (9 female), 10.0 ± 2.3 y XRT: *n* = 17 (6 female), 9.1 ± 2.0 y	N/R	2010 to 2015	Tumor histology: Medulloblastoma, germinoma, glioma, neuroepitelial, neuroblastoma, astrocytoma, neoplasm, craniopharyngioma, glioneural tumor, anaplastic ependymoma, teratoma Tumor location: Infratentorial Supratentorial	General cognitive skills: working memory, and processing speed indices (WISC-IV) Attention: coding test (WISC-IV) Executive function: digit span test (WISC-IV)	↔ Working memory ↔ Processing speed	Total: 4 stars Selection: ** Comparability: – Outcome: **
Weusthof et al. [35]	PBRT: *n* = 26 (10 female), 9.4 (3.2–19.0) y XRT: *n* = 30 (15 female), 9.6 (2.3–17.3) y	N/R	2009 to 2018	Time since diagnosis, mean (range): PBRT: 6.6 (2.0–17.2) y XRT: 9.2 (3.4–16.8) y Total radiation dose, mean (range): PBRT: 51.3 (16.0–74.0) Gy (RBE) XRT: 53.3 (30.0–68.0) Gy (RBE) Tumor histology: Medulloblastoma, glioma, ependymoma, craniopharyngioma, germinoma, others Tumor location: Infratentorial Supratentorial Extraaxial	General cognitive skills: IQ, nonverbal intelligence (CPM and-SPM), working memory, and processing speed indices (WAIS-III, WIE and WISC-IV) Perceptual-motor functions: visual motor integration (Beery-Buktenica Developmental Test) Visuocontructive praxis: visuospatial construction (ROCF) Reasoning: nonverbal reasoning (Raven’s CPM/SPM) Memory/learning: visuospatial memory (ROCF) Attention: coding test (WAIS-III and WISC-IV) Executive function: digit span test (WAIS-III and WISC-IV), categorical and lexical word fluency (WF)	↓ Nonverbal intelligence in XRT ↓ Visuospatial construction in XRT	Total: 5 stars Selection: **** Comparability: – Outcome: *
Yang et al. [31]	PBRT: *n* = 4 (1 female), 5.9 ± 0.5 (5.5–6.4) y XRT: *n* = 4 (2 female), 13.5 ± 3.0 (10.6–16.6) y	N/R	August 2015 to February 2016	Tumor histology: Atyipical meningioma, retinoblastoma, ependymoma, germinoma Tumor location: Upper eyelid, temporal lobre, retina, cerebellum, 4th ventricle, pituitary, suprasellar regions	General cognitive skills: FSIQ, verbal comprehension, perceptual reasoning, fluid reasoning, working memory, processing speed indices (WPPSI-IV and WISC-IV) Memory/learning: memory for design test (NEPSY-II) Attention: coding test (WPPSI-IV and WISC-IV) and CPT-II Executive function: digit span test (WISC-IV), and NEPSY-II animal sorting, design fluency, clocks and inhibition tests	↓ Executive functions with XRT	Total: 3 stars Selection: ** Comparability: – Outcome: *
Yip et al. [27]	PBRT: *n* = 17 (9 female), 5.4 (1.4–17.4)^a^ y XRT: *n* = 32 (6 female), 7.8 (2.4–17.0)^a^ y	Percent of poverty: PBRT: < 13%: 71% ≥ 13%: 29% XRT: < 13%: 53% ≥ 13%: 47% Percent of poverty calculated from patient zip codes via US census data from 2018	1996 to 2019	Total radiation dose, median (range): PBRT: 54 (40–59) Gy XRT: 54 (20–56) Gy Tumor histology: Medulloblastoma, astrocytoma, ependymoma, germinoma, craniopharyngioma, glioma, meningioma, atypical teratoid rhabdoid tumor, germ cell, pineoblastoma, NF-2 associated vestibular schwannoma Time from completion of radiation to assessment, median (IQR): PBRT: 1.8 (0.46–10.6) y XRT: 3.2 (0.11–13.4) y	General cognitive skills: FSIQ, general ability, verbal comprehension, perceptual reasoning, working memory, and processing speed indices (Wechsler) Perceptual-motor functions: visual motor integration (Beery-Buktenica Developmental Test) Memory/learning: verbal learning and memory (CVLT) Attention: coding test (Wechsler) Executive function: forward digit span score (Wechsler), global executive composite, adaptative, externalizing, and internalizing (BRIEF) Adaptive functions: general adaptive composite, abas conceptual domain, and ABAS practical domain (ABAS)	PBRT patients scored higher in all cognitive measures, but treatment modality was not associated with cognitive measures on multivariable regression analyses	Total: 4 stars Selection: ** Comparability: * Outcome: *

Quality was assessed with an adapted form of the Newcastle Ottawa Scale for cross-sectional studies. *ABAS* Adaptive Behavior Assessment System, *BASC* Behavior Assessment System for Children, *Bayley-III* Bayley Scales of Infant and Toddler Development Third Edition, *BRIEF* Behavior Rating Inventory of Executive Function, *BRIEF-P* Behavior Rating Inventory of Executive Function Preschool-Version, *CPM* Raven’s Coloured Progressive Matrices, *CPT* Continuous Performance Test, *CSI* craniospinal irradiation, *CVLT* California Verbal Learning Test, *DAS* Differential Abilities Scales, *DKEFS* Delis–Kaplan Executive Function System, *IQ* intelligence quotient, *FSIQ* full-scale IQ, *IQR* interquartile range, *NEPSY II* A Developmental Neuropsychological Assessment Second Edition, *N/R* not reported, *PNET* primitive neuroectodermal tumors, *PBRT* proton beam radiation therapy, *RIAS* Reynolds Intellectual Assessment Scales, *RBE* relative biological effectiveness accounting for proton irradiation, *ROCF* Rey–Osterrieth Complex Figure Test, *SPM* Raven’s Standard Progressive Matrices, *SB-5* Stanford-Binet Intelligence Scales Fifth Edition, *TB* tumor bed, *WF* Regensburger Word Fluency Test, *WAIS* Wechsler Adult Intelligence Scale, *WASI* Wechsler Abreviated Scale of Intelligence, *WIAT* Wechsler Individual Achievement Test, *WISC* Wechsler Intelligence Scale for Children, *WJ ACH* Woodcock-Johnson Tests of Achievement, *WJ COG* Woodcock-Johnson Tests of Cognitive Abilities, *WMS-IV* Wechsler Memory System Fourth Edition, *WRAML2* Wide Range Assessment of Memory and Learning Second Edition, *XRT* x-ray radiotherapy group

^a^Data are expressed as median (IQR), – not available

*, **, *** a positive point on the Newcastle-Ottawa Scale used for quality assessment

↑ Better results

↔ Similar results

↓ Worse results

### Quality assessment

Two authors (JSM, PLV) independently assessed the methodological quality of the included studies with an adapted form of the Newcastle Ottawa Scale for cross-sectional studies [28]. Studies were given a maximum score of four stars for selection, two stars for comparability, and three stars for outcome. A third author (AL) resolved any potential disagreement.

### Statistical analyses

To minimize the issues found when employing a meta-analytic approach with a small number of studies, we only performed a meta-analysis when a minimum of three studies assessed a given outcome. Pooled mean differences (MD) between groups (expressed as *Z* scores unless otherwise specified, along with 95% CI) were computed using a random effects model (Dersimonian and Laird model) [29]. When two studies shared part of the same sample, only the study with the largest sample was included in the analyses. Begg’s test was used to determine the presence of publication bias, and *I*^*2*^ statistics were used to assess heterogeneity across studies. Sensitivity analyses were performed by removing one study at a time to confirm our results. Sensitivity analyses were also performed by including adjusted data or results from multivariable analyses in studies that reported both nonadjusted and adjusted results. All statistical analyses were performed using Comprehensive Meta-analysis 2.0 (Biostat; Englewood, NJ) setting the level of significance at 0.05.

## Results

### Study selection

From the retrieved articles, 10 studies including 630 survivors of pediatric brain tumors (of whom 53% were treated with PBRT) were included in the systematic review (Supplementary Fig. 1) [24–27, 30–35]. The characteristics of the included studies are summarized in Table 1. Two studies [24, 25] shared part of the same sample, and thus, only the study with the largest sample was included to compute the total number of participants.

### Quality assessment

The quality of the included studies was moderate overall (Table 1). Out of a maximum 10-point score, two studies had a quality score of seven [26, 32], two of six [24, 33], three of five [25, 34, 35], and the remaining studies had a quality score of four or lower [27, 30, 31].

### Study characteristics

The included studies involved between 8 and 150 participants (average of 75 participants) whose average age ranged between 1 and 20 years (Table 1). All studies included both male and female participants (39% of the participants were female).

The most frequently analyzed tumor histologies were craniopharyngioma, medulloblastoma/primitive neuroectodermal tumors, ependymoma, germinoma, astrocytoma, and ependymoma, and the most frequently reported tumor location was infratentorial, followed by supratentorial. The total radiation dose ranged between 30–60 Gy and 20–59 Gy for PBRT and XRT, respectively, and the total craniospinal irradiation (CSI) dose ranged between 15 Gy and 40 Gy for both PBRT and XRT. Most studies reported no differences in major demographic/clinical variables between groups (*e.g*., age, sex, socioeconomic status, tumor histology or location). However, some studies did find differences in some variables, such as tumor location, histology or total radiotherapy dose to the tumor [25, 26, 31, 32, 34].

### Outcomes

**Fig. 1:**
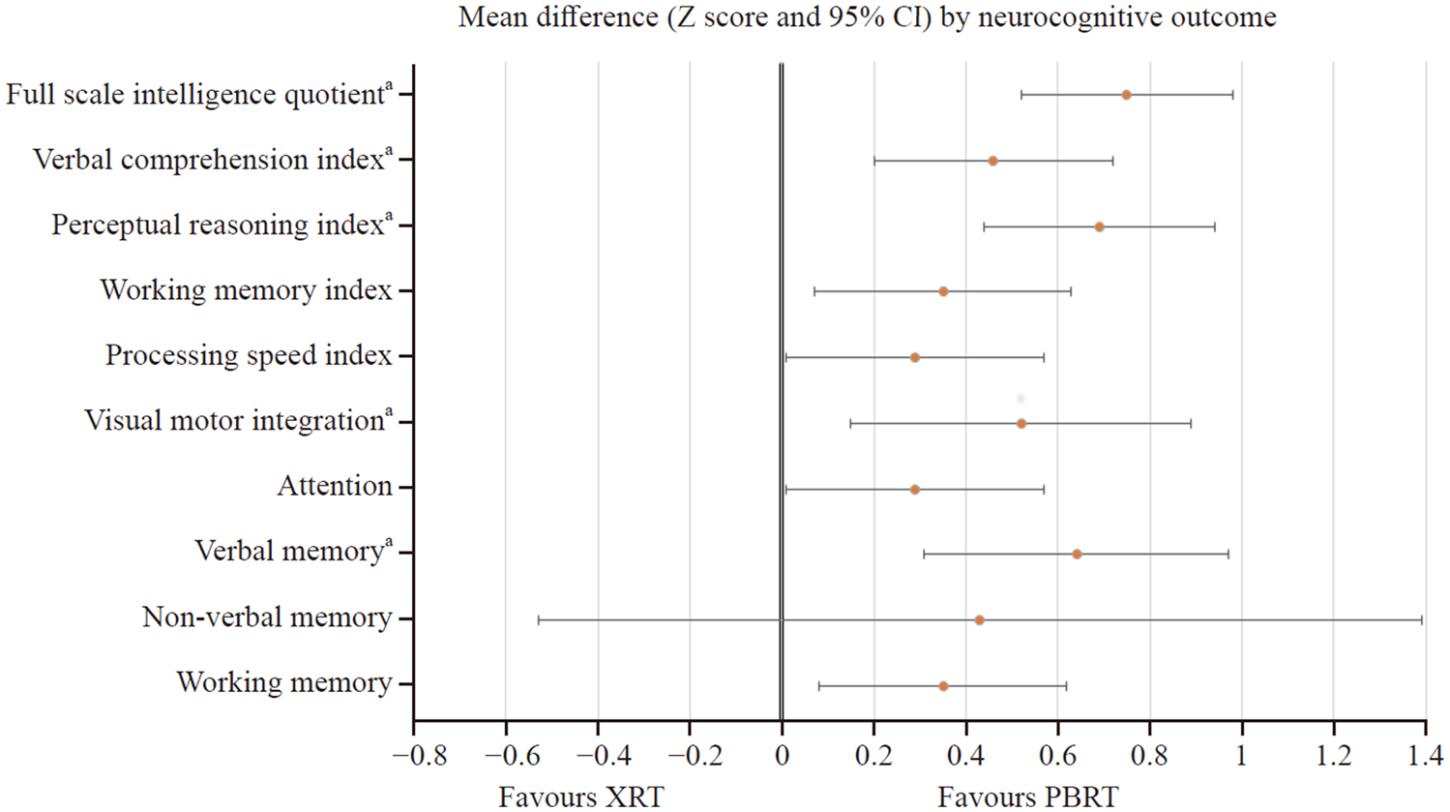
Comparison of neurocognitive outcomes in children and adolescents with brain tumors treated with photon radiation (XRT) or proton therapy (PBRT). ^a^This outcome did remain significant in sensitivity analyses. *CI* confidence interval

A summary of the meta-analyzed outcomes is shown in Table 2. Patients who had received PBRT achieved significantly higher *Z* scores (all *P* < 0.05) than those who had received XRT for most analyzed neurocognitive outcomes, including IQ (evaluated by means of the Full-Scale IQ and other intelligence scores), verbal comprehension, perceptual reasoning and processing speed indices, verbal working memory and working memory index, visual motor integration, focused attention, and verbal memory (Fig. 1). The only domain for which no differences were observed was nonverbal memory (*P* = 0.367). Forest plots for each outcome are available in Supplementary Fig. 2. No signs of heterogeneity (all *I*^*2*^ values < 5%) or risk of bias (all Begg’s *P* values > 0.10) were found for any of the analyzed outcomes.

**Table 2:** Summary of meta-analysis results comparing the neurocognitive outcomes of children and adolescents with brain tumors treated with XRT or PBRT

Outcome	Studies^a^ (participants)	*Z*-score (95% CI)	*P*-value	*I*^*2*^	Begg’s *P*
Full scale intelligence quotient	8 (*n* = 512)	0.75 (0.52, 0.99)	** < 0.001**	0	0.451
Verbal comprehension index	7 (*n* = 385)	0.46 (0.20, 0.73)	**0.001**	0	0.382
Perceptual reasoning index	8 (*n* = 439)	0.69 (0.44, 0.94)	** < 0.001**	0	0.193
Working memory index	9 (*n* = 464)	0.35 (0.07, 0.63)	**0.016**^**b**^	0	0.301
Processing speed index	9 (*n* = 468)	0.29 (0.01, 0.56)	**0.046**^**b**^	0	0.377
Visual motor integration	5 (*n* = 318)	0.52 (0.15, 0.88)	**0.006**	3.5	0.110
Attention	9 (*n* = 468)	0.29 (0.01, 0.57)	**0.044**^**b**^	0	0.377
Verbal memory	4 (*n* = 262)	0.64 (0.31, 0.96)	** < 0.001**	0	0.367
Non-verbal memory	3 (*n* = 144)	0.43 (− 0.53, 1.40)	0.377	0	0.148
Verbal working memory	9 (*n* = 464)	0.35 (0.08, 0.63)	**0.012**^**b**^	0	0.174

Data are shown as *Z*-scores. Significant P-values are in bold font. ^a^The study by Child et al., was counted as two studies as it included two control and interventions groups. ^b^This result did not remain significant in sensitivity analyses. Results statistically significant are shown in bold characters. *CI* confidence interval, *MD* mean difference, *XRT* x-ray radiotherapy group, *PBRT* proton beam radiation therapy

Some studies reported not only nonadjusted data but also adjusted data/multivariable analyses for some outcomes (including different variables in the model such as tumor location, interval between radiotherapy and evaluation, total radiation dose, or CSI dose). Sensitivity analyses were conducted when possible, using adjusted data, and all results remained essentially unchanged except for processing speed index (*P* = 0.089) and focused attention (*P* = 0.163), which became nonsignificant (Supplementary Table 2). Sensitivity analyses by removing one study at a time also confirmed significant differences for most outcomes except for verbal working memory and working memory index, processing speed index and focused attention, which suggests that these results were mostly driven by some individual studies. Despite significant benefits on verbal working memory and working memory index on the overall analysis (*P* = 0.016 and *P* = 0.012, respectively), the results became nonsignificant when removing the study by Kahalley et al. [24], although with a trend toward significance (*P* = 0.072 and *P* = 0.057, respectively). It should be noted that scores extracted from the meta-analyzed papers for these outcomes are heterogeneous, as they include scores from auditory and visual sensory modalities, and this issue may affect the variance of these variables. Sensitivity analysis also showed no consistent benefits for the processing speed index, which despite being significant in the main analysis (*P* = 0.046), became nonsignificant when removing almost every single study (*e.g*., Kahalley et al. [24], Peterson et al. [30], Yip et al. [27], Yang et al. [31], Eaton et al. [33], or Weusthof et al. [35]), except for Gross et al. [34] (*P* = 0.028), and Child et al. [32] (*P* = 0.03). Similarly, focused attention also became nonsignificant when removing the studies of Gross et al. [34], Yip et al. [27], Eaton et al. [33], or Child et al. [32].

Other neurocognitive-related outcomes could not be included in the meta-analyses, as they were assessed by two or fewer studies. These outcomes included the general ability index (Weschler Intelligence Scales), fine motor function (Pegboard Groove), visuoconstructive praxis and memory (Rey-Osterrieth Complex Figure Test), categorical and lexical word fluency (Regensburger Word Fluency Test), executive function (Behavior Rating Inventory of Executive Function and Preschool-Version, Delis-Kaplan Executive Function System and Behavior Assessment System for Children Second and Third Edition), attention [Continuous Performance Test (CPT) Second Edition-II, Behavior Assessment System for Children Second and Third Edition and Bayley Scales of Infant and Toddler Development Third Edition], academic skills (Wechsler Individual Achievement Test, Woodcock-Johnson Tests of Achievement Third and Fourth Edition, and Woodcock-Johnson Tests of Cognitive Abilities), social cognition (Adaptive Behavior Assessment System Second Edition) and adaptative behavior (Adaptive Behavior Assessment System Second Edition). For these outcomes, Gross et al. reported that compared with XRT, PBRT was associated with a higher general ability index with no differences reported for the other outcomes [34].

## Discussion

The main finding of the present systematic review and meta-analysis, which included 10 studies and more than 600 participants, was that patients who received PBRT seem to demonstrate significantly higher scores than those who received XRT on a varied number of neurocognitive outcomes (*i.e.,* full-scale IQ, verbal comprehension, perceptual reasoning and processing speed indices, verbal working memory and working memory index, visual motor integration, verbal memory and focused attention). Sensitivity analyses confirmed significant differences for full-scale IQ, verbal comprehension and perceptual reasoning indices, visual motor integration, and verbal memory.

Numerous variables can affect neurocognitive function in children and adolescents with brain tumors, notably age [36, 37], surgery [35], hydrocephalus [38, 39], chemotherapy [40] or postoperative cerebellar mutism syndrome [41], among others. However, it is well documented that radiation, especially craniospinal radiation, confers the greatest neurocognitive risk [9, 25]. Additionally, regarding radiation, there are many factors that affect the neurocognitive development of these patients, such as radiation field, focal/CSI, boost volume or CSI dose. In this regard, although modern XRT techniques that enable tighter conformality of the administered dose around targets [*e.g.*, intensity-modulated radiotherapy (RT), tomotherapy, etc.] seem to have improved intellectual benefits [42–44], these benefits do not seem to yet be as significant as those with PBRT [45]. All patients in this meta-analysis who received photon therapy were treated after 2000 with modern techniques.

In line with our findings, a recent systematic review on cognitive changes following PBRT or XRT in pediatric brain tumor patients found significantly poorer cognitive outcomes—particularly worse general cognition and working memory—among patients treated with XRT compared with PBRT [45]. Craniospinal irradiation was consistently associated with poorer cognitive outcomes, while focal therapy was associated with minor cognitive changes [45]. However, to the best of our knowledge, this is the first meta-analysis that quantitatively compares the neurocognitive outcomes of pediatric survivors of brain tumors after treatment with XRT or PBRT.

The most homogeneous study included in the present meta-analysis evaluated patients with medulloblastoma treated contemporaneously on comparable treatment protocols that differed only in RT modality (PBRT or XRT) [24]. This study revealed significantly different scores between the PBRT and XRT groups in global IQ, perceptual reasoning and working memory indices favoring the PBRT group [24]. At four years after RT, patients treated with PBRT exhibited overall stable performance over time in all neurocognitive domains except for the processing speed index. In contrast, patients treated with XRT exhibited a significant decline in global IQ, working memory and processing speed scores [24]. Even in the context of CSI, patients treated with PBRT showed stable intellectual outcomes in most domains and experienced significantly better long-term outcomes in global IQ, perceptual reasoning and working memory indices compared with patients treated with XRT [24]. These findings were also confirmed by Eaton et al. in very homogeneous standard-risk medulloblastoma patients matched 1:1 based on demographic and clinical characteristics [33]. Patients treated with PBRT demonstrated higher scores for intelligence after treatment than their counterparts treated with XRT, with the former scoring about 1.5 SD (between 22 and 23 points) higher than the XRT group for FSIQ, verbal and nonverbal outcomes.

The remaining studies included in the present meta-analysis involved different pediatric brain tumors. Kahalley et al. [25] compared the IQ scores of 150 patients (90 receiving PBRT) with different tumor histologies. In the PBRT group, no change in IQ over time was identified, whereas in the XRT group, IQ declined by 1.1 points per year. IQ was lower in the XRT group (by 8.7 points) than in the PBRT group. Among the 82 patients treated with CSI, FSIQ was 12.5 points lower in the XRT group than in the PBRT group, and although IQ remained stable over time among PBRT patients, IQ decreased, on average, by 1.57 points per year in the XRT patients. Gross et al. [34] compared neuropsychological outcomes of different brain tumor histologies in 125 patients who underwent XRT or PBRT. On multivariable analysis, PBRT was associated with higher full-scale IQ and processing speed index relative to XRT, with a trend toward higher verbal IQ and general adaptive functioning. Weusthof et al. [35] evaluated neurocognitive outcomes in 56 pediatric brain tumor patients who received PBRT *vs*. XRT. There were no alterations in long-term neurocognitive abilities after PBRT, whereas declines in the processing speed index, nonverbal intelligence, and visuospatial abilities were observed after XRT.

In the study by Child et al. [32], patients treated with focal PBRT scored within normal limits on most cognitive measures and generally performed comparably to normative samples of typically developing children. Only mild challenges in processing speed index, fine motor, and academic fluency skills were seen in this cohort. The focal XRT cohort showed worse results than expected for age on global intellectual functioning. This study also confirmed that CSI radiation confers the greatest neurocognitive risk [9, 25]. After a long follow-up, the CSI XRT group was severely impaired, with 76% of the patients showing clinically impaired global intellectual functioning and 53%–88% demonstrating impaired performance across all cognitive and academic fluency tasks.

The processing speed index has been reported as the most vulnerable domain regarding neurocognitive outcome in pediatric brain tumor patients [24, 32, 46–48]. This domain shows a decrease in longitudinal development with a below-average IQ in patients treated with surgery only, XRT or PBRT [35]. Processing speed depends on intact white matter connections, and its tasks reflect both cognitive efficiency and fine motor functioning. White matter tracts can be harmed by surgery or radiation [49–51]. Interestingly, in three out of the nine studies meta-analyzed for this domain, patients receiving PBRT experienced significantly less processing speed index decline when compared to XRT.

Socioeconomic status (SES) has been demonstrated to be a predictor of cognitive outcomes for pediatric brain tumor patients both at treatment initiation and over time. Higher SES appears to serve as a protective factor mitigating the harmful effects of treatment on cognitive functioning. SES may represent a useful focal point for improving interventions, as those in low SES groups may be better served through broad policy change, education, and support [52]. In some countries, proton therapy is only available to patients with certain types of insurance or with wealth to be able to pay for the treatment (and travel to a proton center if there isn´t one nearby). SES could not be meta-analysed, as only three out of the nine studies had taken this factor into account but with different methods of assessing it.

A major strength of the present meta-analysis is that, to our knowledge, it is the first to compare the effects of PBRT and XRT on neurocognitive outcomes in children and adolescents with brain tumors. Several limitations must, however, be acknowledged, notably the small number of available studies, which impeded us from performing subanalyses attending to major variables, including patient (*e.g*., age, tumor histology or location, socioeconomic status) and treatment characteristics [*e.g*., treatment dose, timing, modality of radiotherapy (focal or CSI)]. Indeed, several studies did not adjust for these clinical/descriptive variables in their analyses, which might be viewed as a potential bias and would also affect the present results. It must be noted, however, that we attempted to perform sensitivity analyses by including adjusted data when available, and the results were confirmed. The heterogeneity found in the methods used for the assessment of neurocognitive outcomes can also be considered a limitation, as well as the heterogeneity found in participants’ characteristics. To bypass this limitation, we analyzed all outcomes as *Z* scores instead of absolute scores, although the latter could have provided more accurate information. Moreover, data from some studies could not be meta-analyzed because the necessary data were not available despite contacting the corresponding authors, which might be regarded as a potential bias.

Despite the abovementioned limitations, the present meta-analysis has relevant clinical implications. Our findings highlighted the potential of PBRT for the improvement of long-term psychosocial functioning in adult survivors of pediatric brain tumors by mitigating multiple neuropsychological sequelae of radiation treatment. It must be noted, nonetheless, that although patients treated with PBRT may have less neurocognitive impairment than those treated with XRT, the former are vulnerable to post-RT side effects, and therefore, these patients should also be closely monitored and encouraged to participate in interventions aimed at improving their neurocognitive functioning.

In conclusion, patients who have received PBRT achieve significantly higher scores on most analyzed neurocognitive outcomes (including IQ, verbal comprehension and perceptual reasoning indices, visual motor integration, and verbal memory) than those who have received XRT. These results can be used to guide treatment planning and indicate targets for monitoring and neurocognitive intervention. Future high-quality research is warranted to identify how patient (*e.g*., age, tumor histology or location) and treatment characteristics [*e.g*., treatment dose, timing, modality of radiotherapy (focal or CSI)] might affect neurocognitive outcomes of children and adolescents with brain tumors treated with PBRT. Larger studies with long-term follow-ups are needed to confirm these results.

## Supplementary Information

Below is the link to the electronic supplementary material.

Supplementary file1 (ESM 1041 KB)

## Data Availability

Data are available upon request.
