# Supplementary material for: Neurocognitive outcomes in pediatric brain tumors after treatment with proton versus photon radiation: a systematic review and meta-analysis
Source: World J Pediatr. 2023 May 8;19(8):727–40. doi: 10.1007/s12519-023-00726-6 (PMC10348930; doi:10.1007/s12519-023-00726-6)

**Supplementary Table 1.** Search Strategy for PubMed.

| **1.** | proton |
| --- | --- |
| **2.** | child* |
| **3.** | pediatric |
| **4.** | paediatric |
| **5** | infant |
| **6** | adolescen* |
| **7** | 2 OR 3 OR 4 OR 5 OR 6 |
| **8.** | 1 AND 7 |

Filters: Title and abstract.

- PubMed was searched from inception to February 1, 2022.

**Supplementary Table 2.** Sensitivity analyses for those outcomes in which some results reported adjusted data/multivariate models.

| Outcome | Studies*  (participants) | SMD (95%CI) | *p*-value | *I2* | *Begg’s p* |
| --- | --- | --- | --- | --- | --- |
| Full scale intelligence quotient | 8 (n = 512) | 0.41 (0.23, 0.59) | **<0.001** | 0 | 0.087 |
| Verbal comprehension index | 7 (n = 385) | 0.30 (0.10, 0.51) | **0.004** | 0 | 0.381 |
| Perceptual reasoning index | 8 (n = 439) | 0.43 (0.18, 0.69) | **0.001** | 0 | 0.087 |
| Working memory index | 10 (n = 464) | 0.21 (0.03, 0.38) | **0.022** | 0 | 0.296 |
| Processing speed index | 9 (n = 468) | 0.18 (-0.03, 0.38) | 0.089 | 0 | 0.301 |
| Attention | 10 (n = 468) | 0.12 (-0.05, 0.29) | 0.163 | 0 | 0.186 |

Results are shown as standardized mean difference (SMD) along with 95% confidence intervals (CI), as not enough data were available for the computation of Z-scores in some studies. Significant p-values are in bold font. *The study by Child et al. was counted as two studies as it included two control and interventions groups.


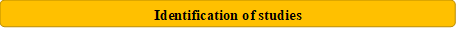


Records identified from:

- Pubmed (n = 2,974)

- Web of Science (n = 12,440)

- Cochrane (n = 717)

- Embase (n = 5,301)

**Identification**

Records screened

(n = 21,432)

Records excluded

(n = 21,407)

**Screening**

Reports not retrieved

(n = 0)

Reports sought for retrieval

(n = 25)

Reports excluded:

- did not assess cognitive-related outcomes (n = 6)

- analyzed prediction models or used mathematical models (n = 5)

- did not compare proton therapy *vs* photon radiation (n = 4)

Reports assessed for eligibility

(n = 25)

Studies included in review

(n = 10)

**Included**

Studies excluded:

- incomplete data (n = 1)

Studies included in meta-analysis (n = 9)

**Supplementary Figure 1**. Flow diagram of literature search.

**Supplementary Figure 2.** Forest plots for each outcome.


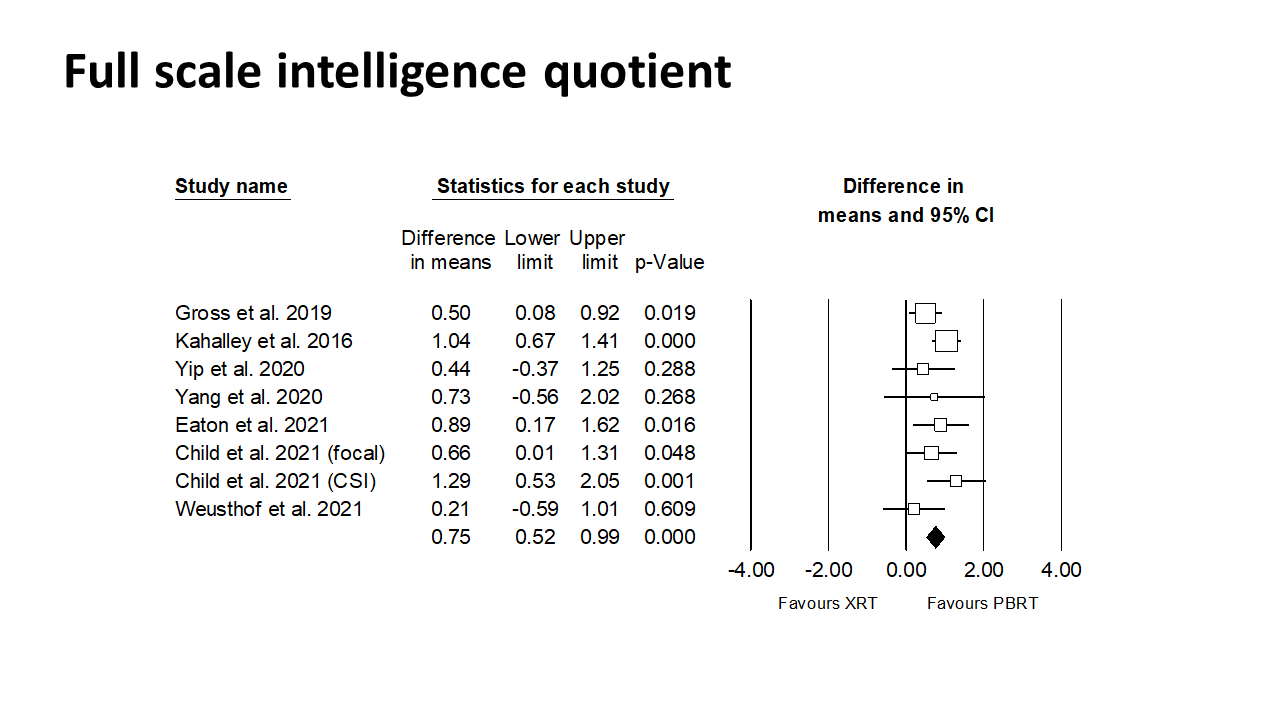


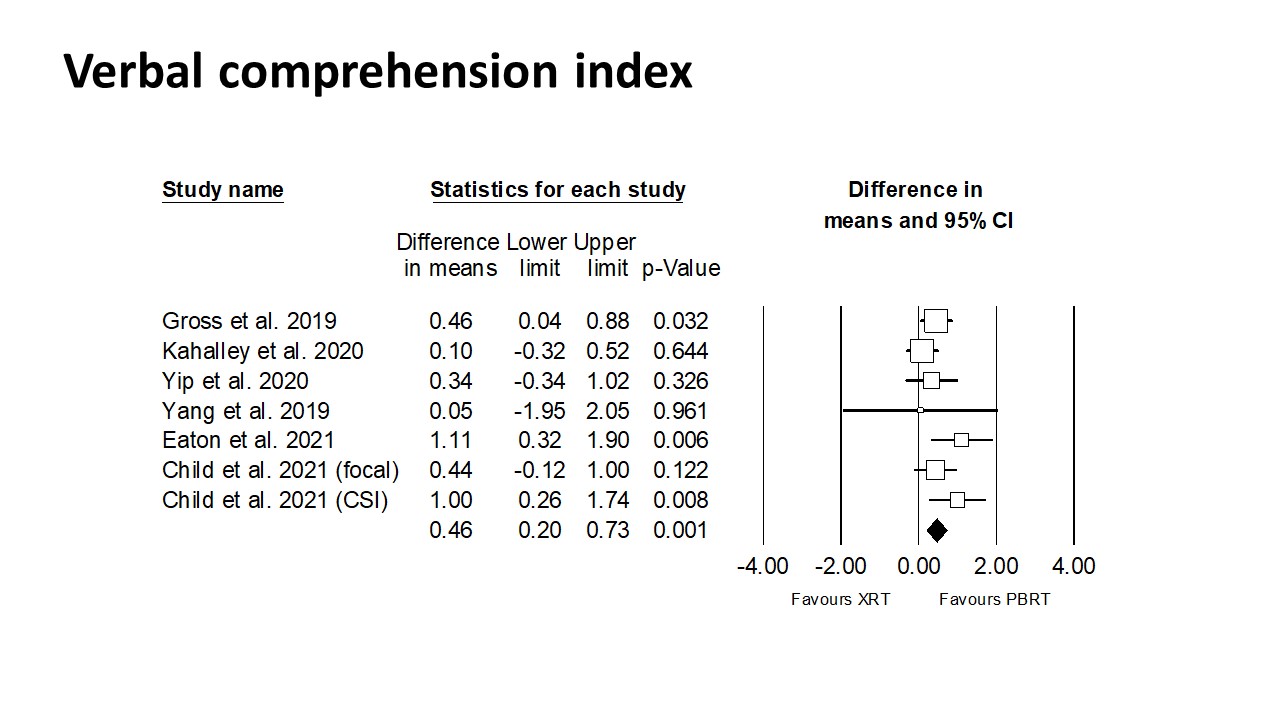


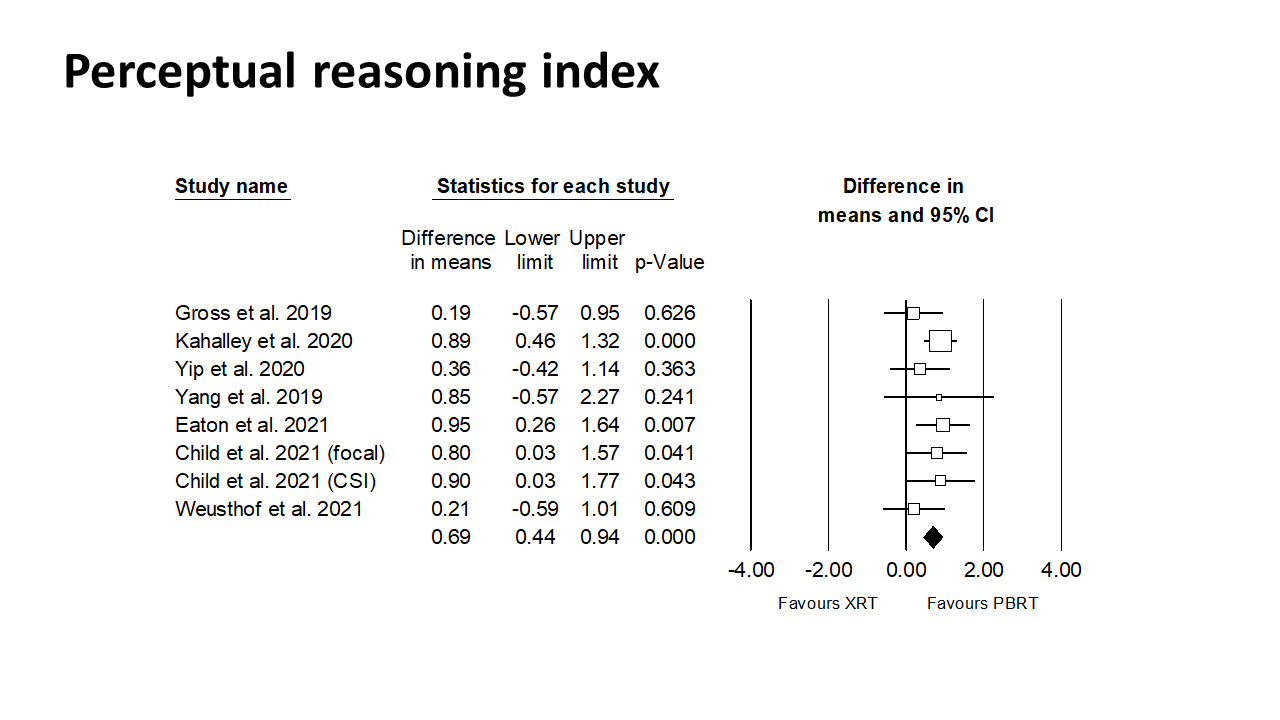


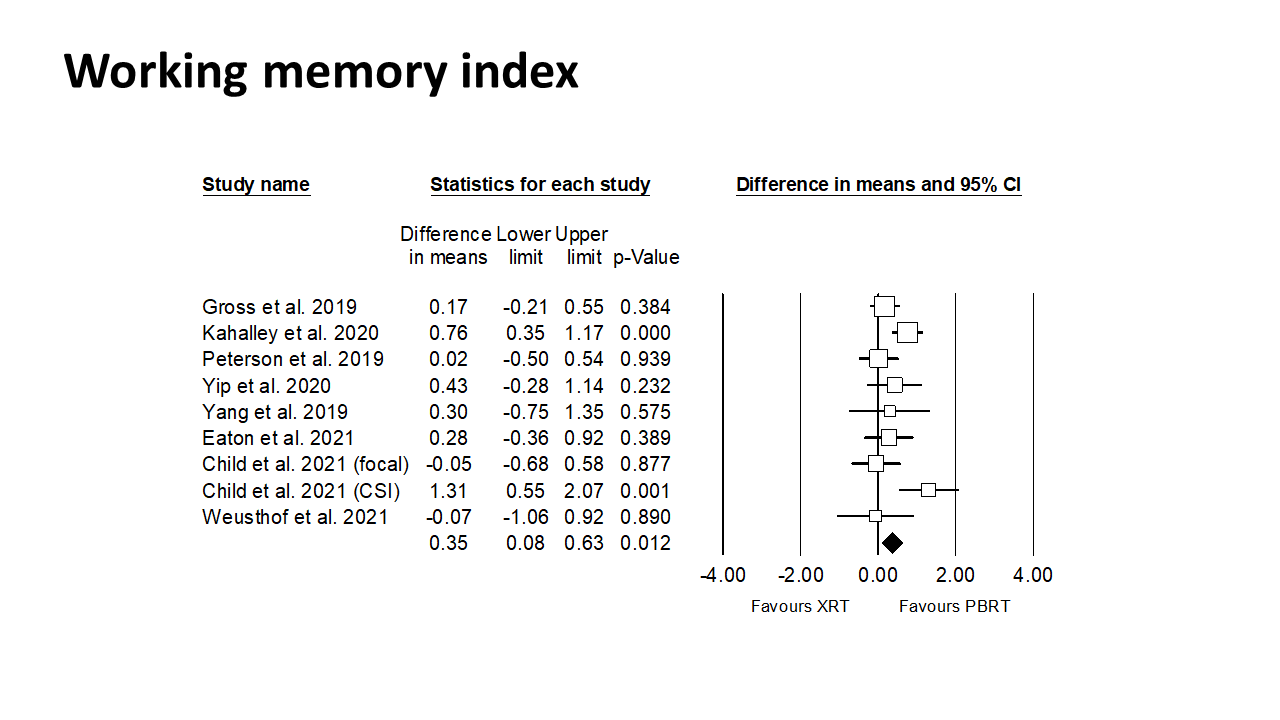


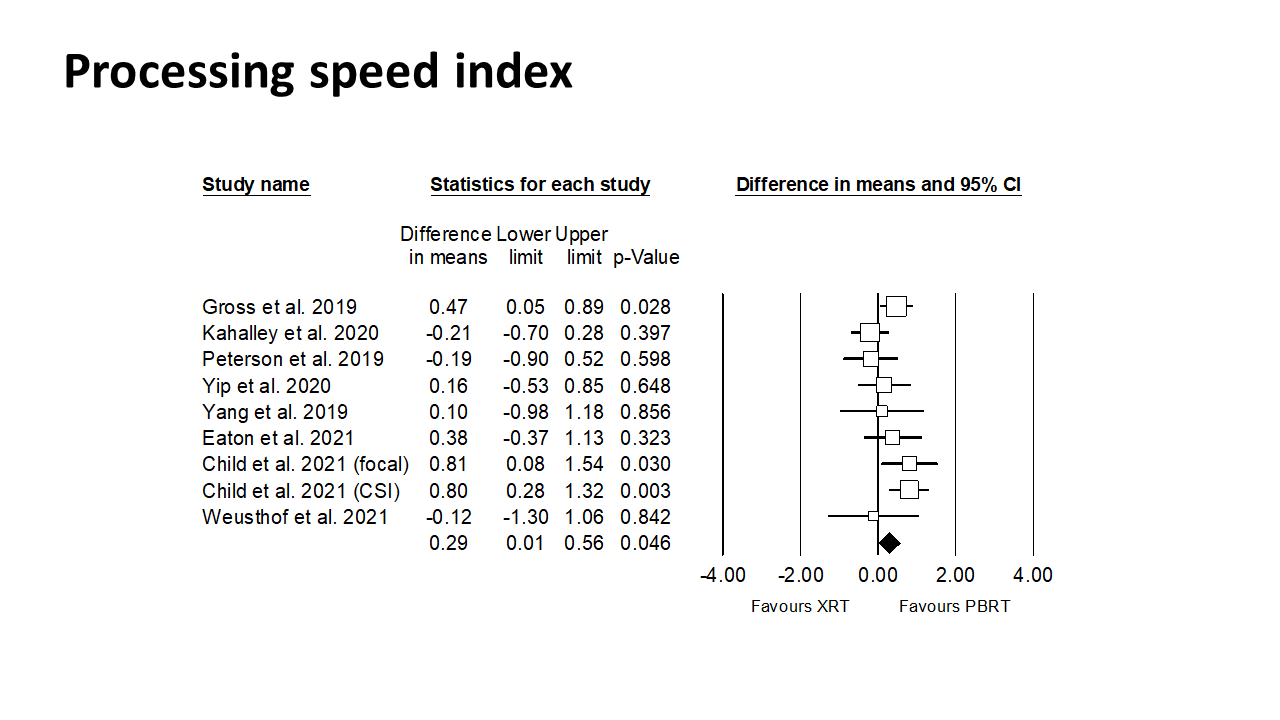


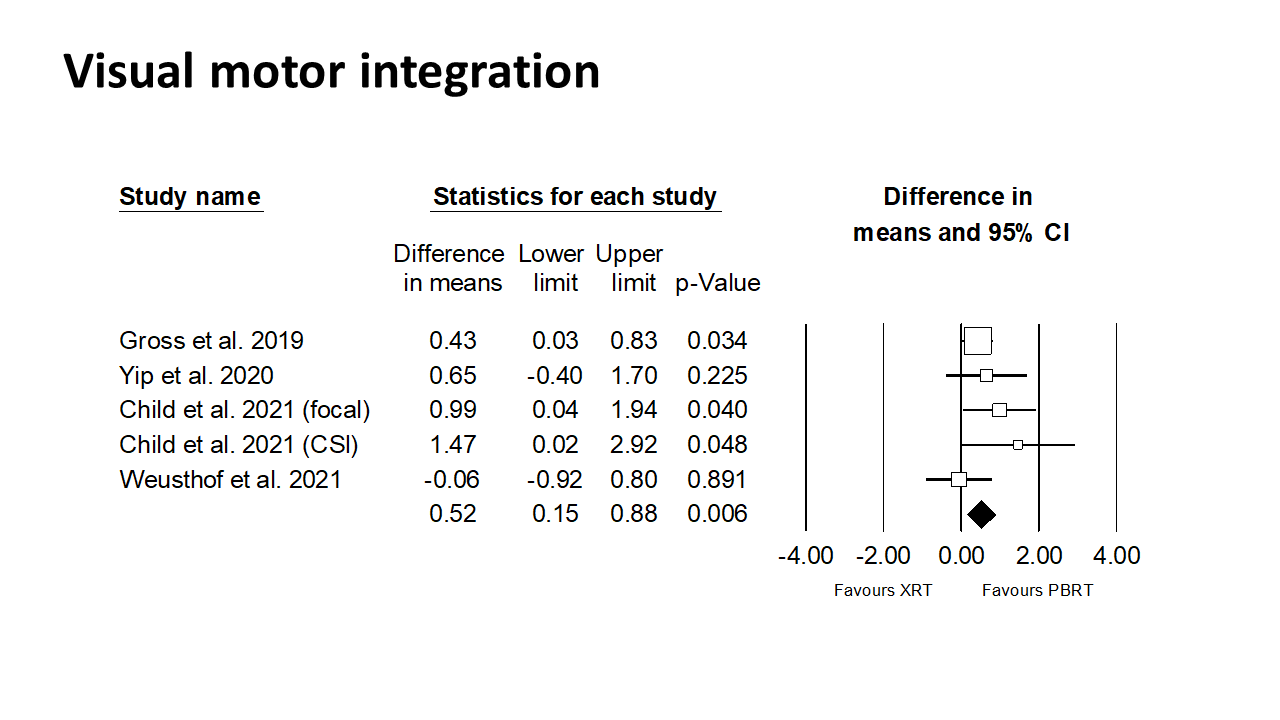


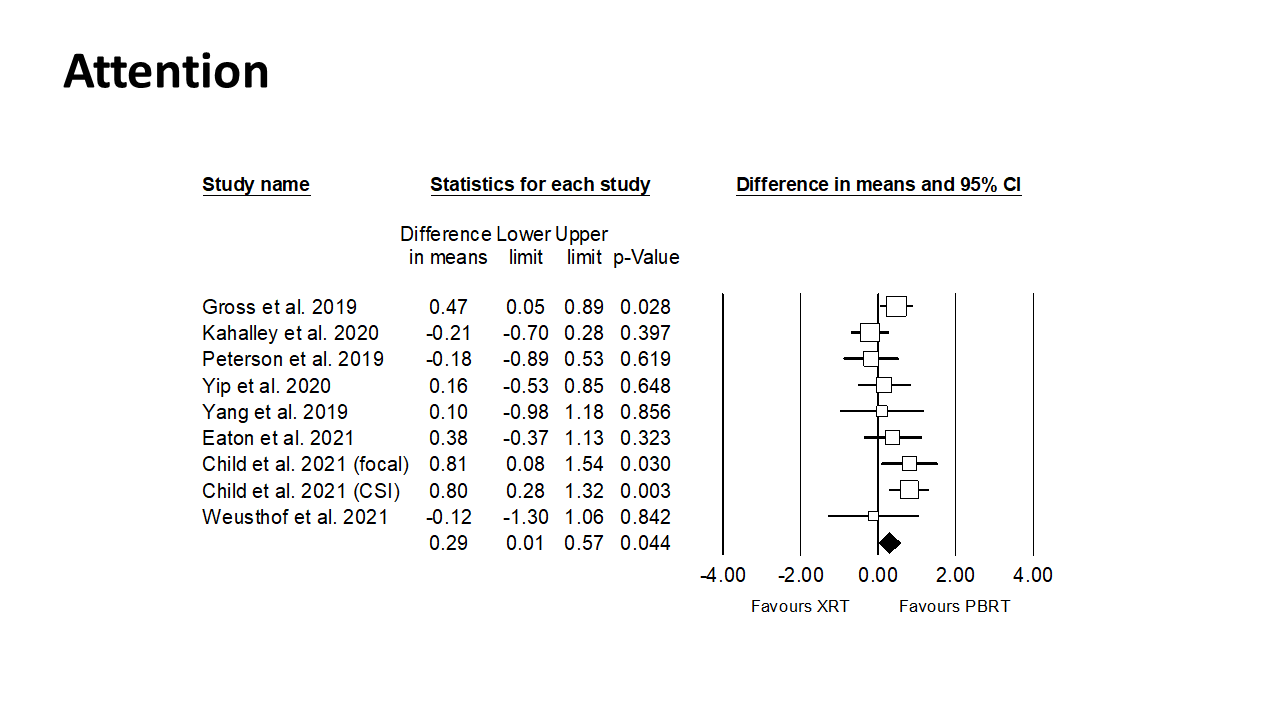


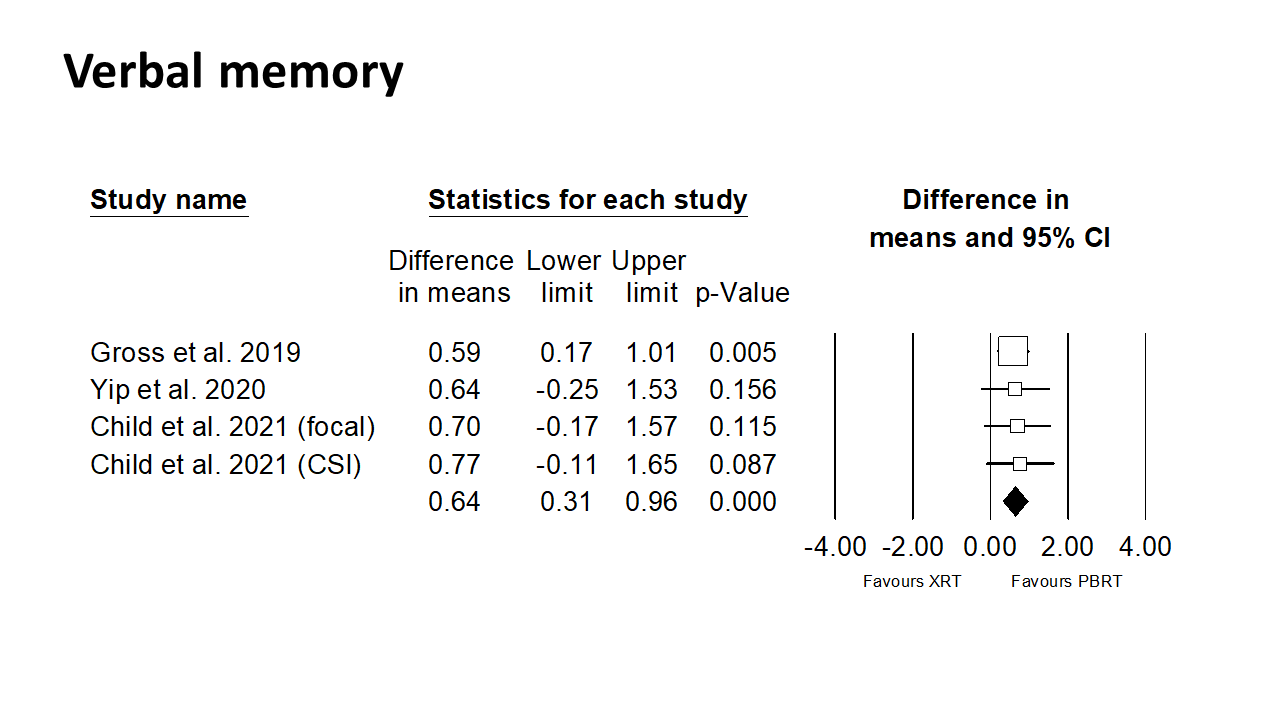


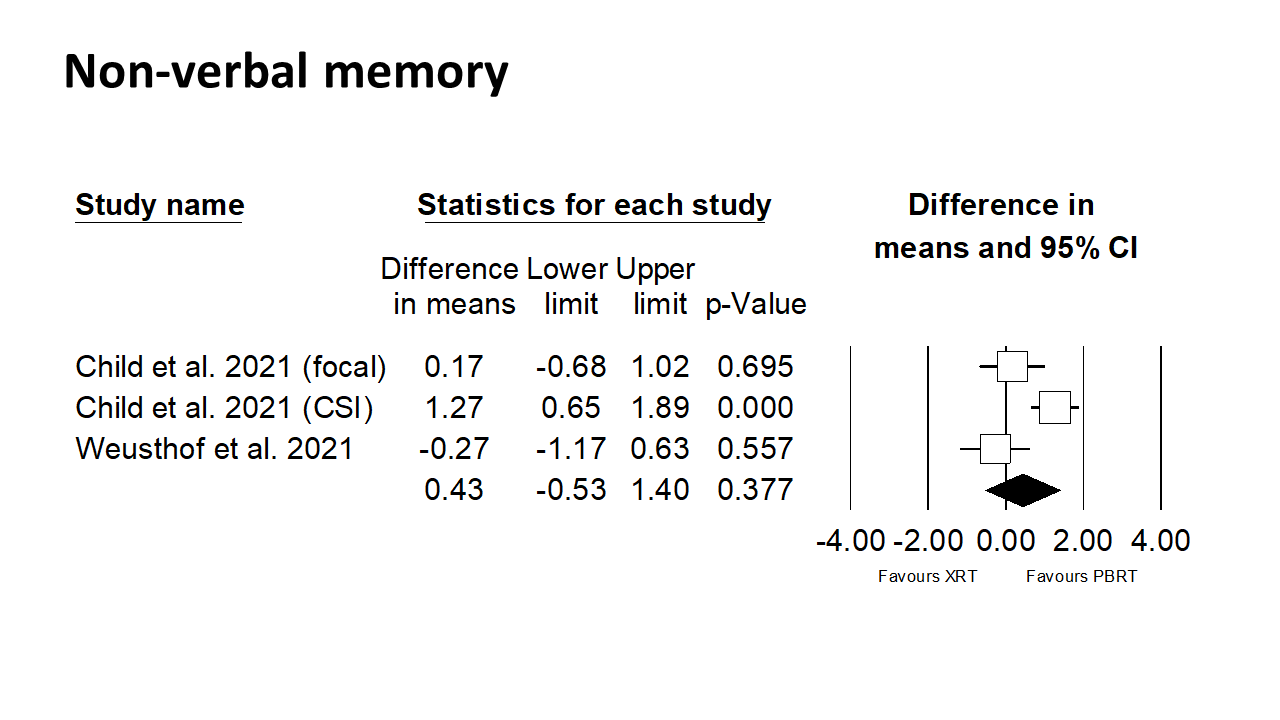


**Working memory**


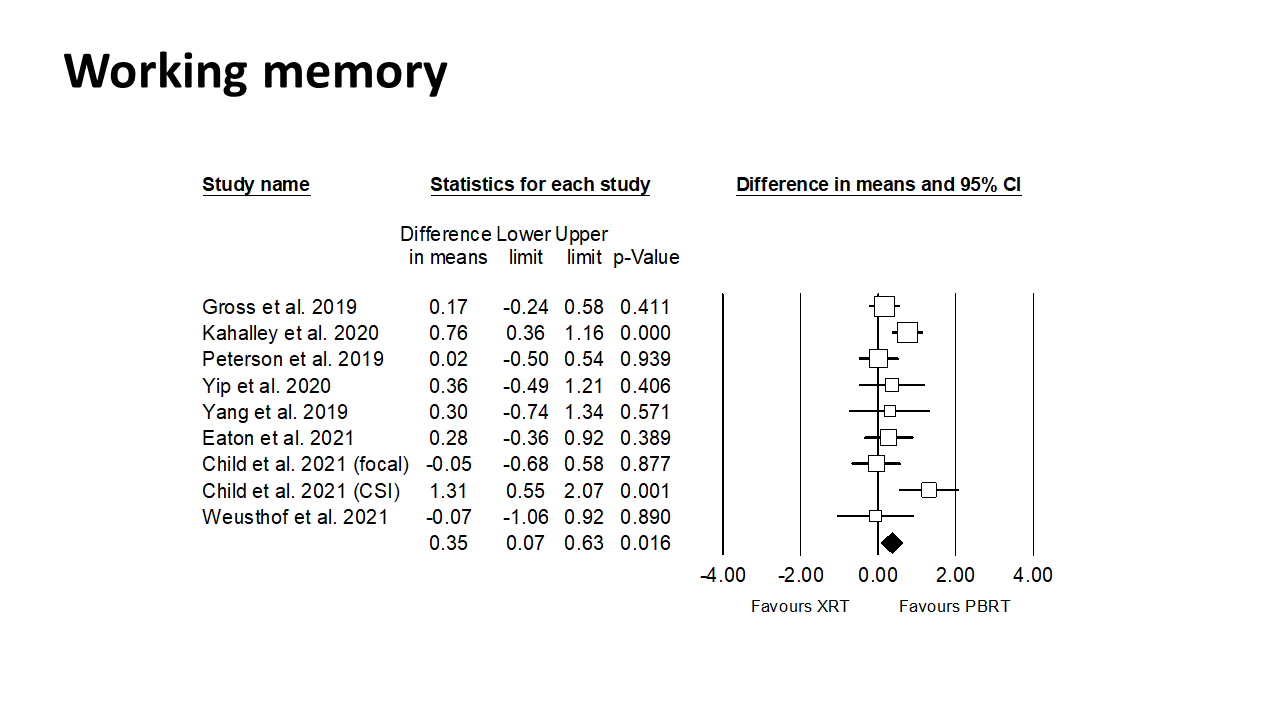

Supplement: Supplementary file 1 — Supplementary file1 (ESM 1041 KB) [file 12519_2023_726_MOESM1_ESM.docx]
